# Longitudinal change in central corneal thickness among primary schoolchildren

**DOI:** 10.1016/j.optom.2025.100537

**Published:** 2025-03-02

**Authors:** Hassan Hashemi, Mehdi Khabazkhoob, Elham Azizi, Mohammad Hassan Emamian, Akbar Fotouhi

**Affiliations:** a Noor Research Center for Ophthalmic Epidemiology, Noor Eye Hospital, Tehran, Iran; b Department of Basic Sciences, School of Nursing and Midwifery, Shahid Beheshti University of Medical Sciences, Tehran, Iran; c Department of Optometry and Vision Science, University of Melbourne, Melbourne, Australia; d Ophthalmic Epidemiology Research Center, Shahroud University of Medical Sciences, Shahroud, Iran; e Department of Epidemiology and Biostatistics, School of Public Health, Tehran University of Medical Sciences, Tehran, Iran

**Keywords:** Corneal thickness, Children, Cohort study, Ocular biometry

## Abstract

**Purpose:**

To investigate the longitudinal changes in corneal thickness and its contributing factors in primary schoolchildren.

**Methods:**

This study is a part of the Shahroud Schoolchildren Eye Cohort Study, conducted longitudinally in two phases; in 2015 and then in 2018. Participants were tested for uncorrected visual acuity, best-corrected visual acuity, cycloplegic refraction, biometry, and Scheimpflug corneal imaging.

**Results:**

After applying the exclusion criteria, 8782 eyes from 4432 participants were analysed. Of these, 2309 (52.1%) were male. Average three-year changes in central corneal thickness (CCT) and corneal apex thickness were −0.58 (95% CI: −0.94 to −0.22) and −0.59 (95% CI: −0.95 to −0.23) microns, respectively. There was an increase of 8.63 (95% CI: 8.1 to 9.17) microns in the superior corneal thickness after three years while the inferior corneal thickness decreased by an average of −5.75 (95% CI: −6.3 to −5.2) microns. The multiple generalized estimating equation (GEE) model showed that the three-year changes in the CCT were lower in rural than in urban students (β = −1.71; *p* < 0.001). Moreover, the baseline CCT (β = −0.04; *p* < 0.001), anterior chamber depth (β = −1.6; *p* = 0.003), and corneal diameter (β = −1.18; *p* < 0.001) had a significant inverse association while the body mass index (β = 0.12; *p* = 0.002) and axial length (β = 0.84; *p* < 0.001) had a significant direct association with the 3-year changes in CCT. After three years, CCT decreased by advancing age in boys, while remaining almost constant in girls.

**Conclusion:**

The 3-year change in CCT was clinically negligible and could indicate its stability in schoolchildren. However, CCT thinning was more remarkable in urban students.

## Introduction

Corneal thickness is an important parameter in assessing corneal morphology and can provide valuable information on corneal changes caused by abnormal conditions such as ectatic disease, corneal hypoxia, or edema following contact lens wear or at high altitudes.^1^, ^2^, ^3^, ^4^ Central corneal thickness (CCT) is a crucial factor in the accurate measurement of intraocular pressure (IOP).^5^^,^^6^ Furthermore, CCT evaluation before and after refractive surgery is important for assessing the surgical outcome.^7^, ^8^, ^9^, ^10^

The distribution of CCT has been extensively investigated in adults and among different ethnicities,^11^, ^12^, ^13^, ^14^, ^15^, ^16^, ^17^ but there is sporadic and inconsistent data on the children's population. A study by the Pediatric Eye Disease Investigator Group (PEDIG)^18^ on 2079 children between 1–17 years showed that the CCT increases slightly with age with the majority of changes occurring between 1–11 years. Zhang et al.^19^ failed to find any significant association between age and CCT in 926 children aged 8–16 years, possibly due to not including younger ages than 8 years. The results of a large-scale study evaluating CCT in 4956 Iranian children between 6 and 12 years were contradictory, with CCT showing a decrease from 6 to 12 years.^20^ Evidence suggests that ectatic conditions like keratoconus (KCN) progress more rapidly in children and are also more severe at the time of diagnosis.^21^^,^^22^ In a retrospective interventional cohort study, a progression rate of 88% was observed among 59 eyes of 49 KCN children aged 9–19 years.^23^ In a retrospective study of 216 adult and children, KCN was more severe at the time of diagnosis in children under 16 years old compared to adults.^24^ Lower thinnest corneal thickness, higher average central corneal keratometry, increased posterior elevation, frequent eye rubbing, and allergic eye disorders have been suggested as risk factors for pediatric keratoconus.^21^ Therefore, understanding the changes in corneal thickness in children can assist in identifying children at risk and initiating management strategies at a younger age in an efficient manner. In this longitudinal study, we investigated the changes in CCT in children between 6 and 12 years old. To the best of our knowledge, this is the first study to assess the longitudinal changes in CCT among children. Moreover, sex, living place, body mass index (BMI), and axial length (AL) at baseline were also examined as potential contributing factors for CCT changes.

## Methods

This report is a part of the Shahroud Schoolchildren Eye Cohort Study. The study's target population was urban and rural students of Shahroud City, northeast Iran. The first phase of this study was conducted in 2015; its methodological details have already been published,^25^ and the second phase was conducted in 2018. Due to the limited number of rural students, all of them were invited for this study, while a multi-stage stratified cluster sampling was performed for urban students. Each classroom was defined as a cluster and a total of 200 from 473 available clusters in Shahroud city were selected. In the second phase, all the participants in the first phase were invited. At the examination room, first, the purpose and protocol of the study were explained to the student's parents or guardians, and a signed consent form was obtained. Then, the medical and demographic records were collected through an interview. After that, optometric examinations and ocular imaging were conducted.

The uncorrected distance visual acuity (UCDVA) was measured using the Nidek CP-770 chart projector (Nidek Co. Ltd, Gammagori, Aichi, Japan) at 3 m (m). Then, non-cycloplegic objective refraction was performed using an autorefractometer (ARK-510A, Nidek Co, Aichi, Japan). The autorefraction results were refined using the Heine Beta 200 retinoscope (Heine Optotechnik, Herrsching, Germany). Finally, all students with a UCDVA worse than 20/20 underwent subjective refraction.

Ocular imaging in this study included corneal imaging, biometric measurements, and retinal imaging. Corneal imaging was carried out using Pentacam HR (Oculus, Inc., Lynnwood, WA). This device works based on the Scheimpflug photography principle and provides about 138,000 data points from the anterior ocular segment in less than 2 s. Oculus software No. 6.10r56/1.25r15 was used. All ocular examinations (both eyes) were conducted between 8 a.m. and 4 p.m., at least 2 h after waking up to account for diurnal variation. Ocular biometry was performed using Allegro Biograph (WaveLight AG, Erlangen, Germany). Finally, cycloplegic refraction was conducted using cyclopentolate 1%; two drops were instilled 5 min apart, and cycloplegic autorefraction was performed 30 min after the last drop. The study location and setting in the second phase were similar to the first phase.

### Exclusion criteria

Exclusion criteria were any history of ocular surgery, using contact lenses during the study period, missing data or erroneous data, and best-corrected distance visual acuity (BCDVA) worse than 20/32. Moreover, students suspected of keratoconus were excluded from this report.

### Definitions

Refractive errors were defined based on the spherical equivalent (SE) of cycloplegic refraction. A SE equal to or worse than −0.50 diopters (D) was defined as myopia, and a SE equal to or worse than +2.00 D was considered hyperopia.^26^

#### Statistical analysis

The mean and 95% confidence interval (CI) of corneal thickness in the center and different regions of the cornea were reported. To show the three-year changes in corneal thickness, we reported the difference in corneal thickness values between phases 1 and 2. To better show the distribution of corneal thickness changes over three years, interquartile range (IQR) and 95% and 99% percentiles of changes were also reported by age and sex. To calculate standard error, the cluster sampling method was considered in the analysis and the sampling weight was taken into account according to the sampling method. Since the results of both eyes were analyzed, simple and multiple generalized estimating equation (GEE) models were used to evaluate associations between corneal thickness changes and the study variables.

#### Ethical considerations

The study was conducted in accordance with the Helsinki Declaration. All procedures involving children were approved by the Ethics Committee of Shahroud University of Medical Sciences. Written informed consent was obtained from the students’ parents/legal guardians and oral consent was obtained from all students.

## Results

In this study, 8782 eyes from 4432 children were analyzed. Of those, 2309 (52.1%) were male and the mean age of the participants was 9.67 ± 1.68 years. Table 1 presents the descriptive statistics for various biometric indices and refractive errors categorized by baseline age.

**Table 1:** The mean and standard deviation (SD) for various biometric indices and refractive errors categorized by baseline age.

		AL	Mean-K	ACD	LT	CD	SE
Age(year)	n	mean ± SD	mean ± SD	mean ± SD	mean ± SD	mean ± SD	mean ± SD
6	190	22.62 ± 0.72	43.51 ± 1.53	2.93 ± 0.24	3.58 ± 0.19	12.26 ± 0.44	1.09 ± 0.75
7	687	22.67 ± 0.69	43.49 ± 1.45	2.94 ± 0.23	3.56 ± 0.19	12.23 ± 0.44	1.09 ± 0.64
8	822	22.87 ± 0.68	43.45 ± 1.43	3.01 ± 0.24	3.51 ± 0.2	12.29 ± 0.45	0.96 ± 0.74
9	840	22.99 ± 0.72	43.37 ± 1.38	3.03 ± 0.24	3.48 ± 0.18	12.28 ± 0.45	0.94 ± 0.78
10	702	23.19 ± 0.7	43.17 ± 1.42	3.07 ± 0.24	3.45 ± 0.18	12.32 ± 0.43	0.83 ± 0.85
11	716	23.22 ± 0.73	43.27 ± 1.39	3.1 ± 0.23	3.45 ± 0.18	12.32 ± 0.44	0.76 ± 0.92
12	475	23.32 ± 0.74	43.29 ± 1.44	3.12 ± 0.24	3.44 ± 0.18	12.32 ± 0.44	0.58 ± 0.8

AL, Axial length; mean-K, Mean keratometry; ACD, Anterior chamber depth; LT, Lens thickness; CD, Corneal diameter; SE, Spherical equivalent.

**Fig. 1:**
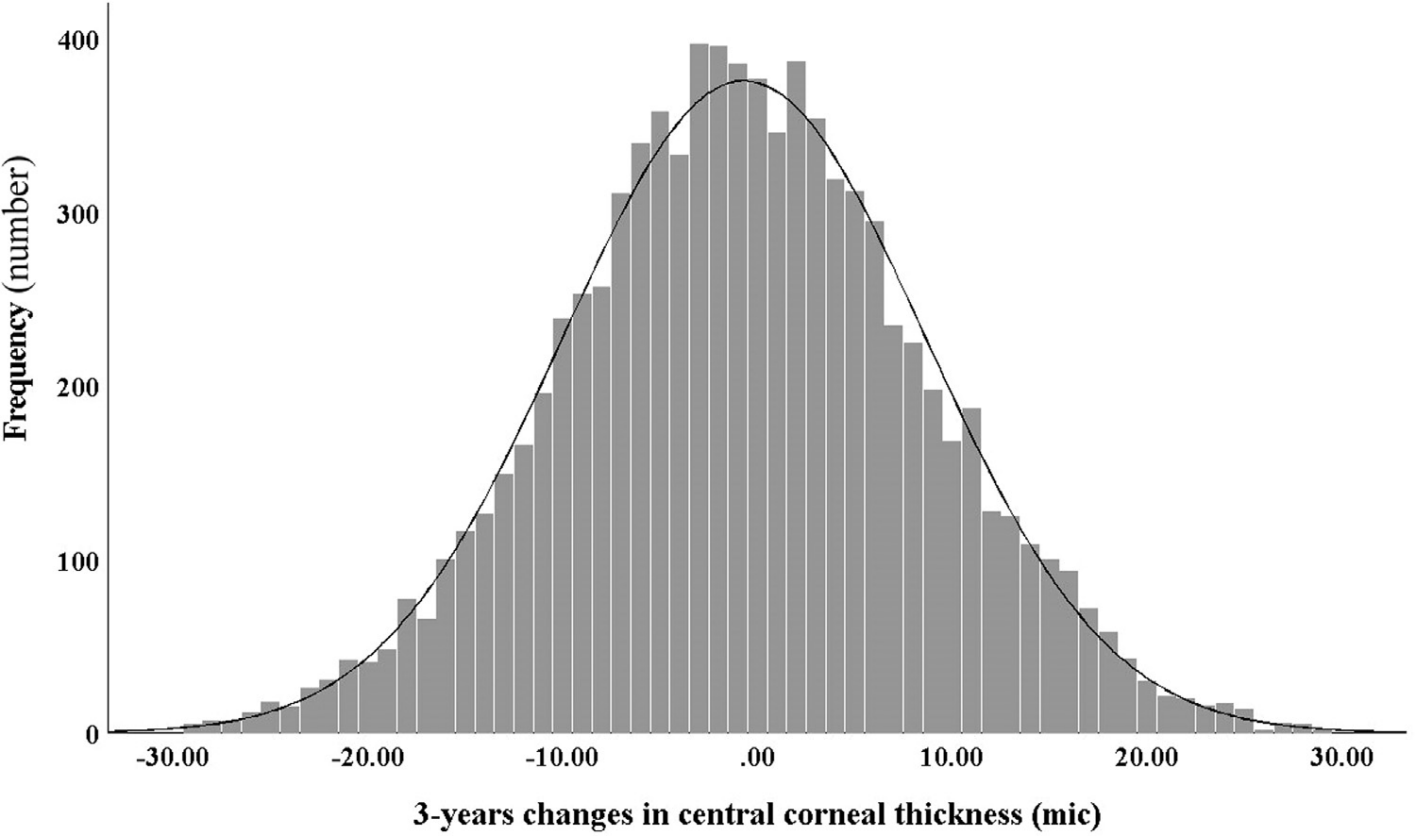
Distribution of 3-year change in central corneal thickness among schoolchildren between 6 and 12 years.

Table 2 shows the mean and 95% CI of corneal thickness in phase 1 and phase 2 in the center and different corneal regions in all participants distributed by sex and age. Fig. 1 illustrates the distribution of 3-year changes in CCT. Skewness and kurtosis of the 3-year changes in corneal thickness were 0.026 and −0.041 µm, respectively. The mean three-year changes in the corneal thickness in the center and apex point were −0.58 (95% CI: −0.94 to −0.22) and −0.59 (95% CI: −0.95 to −0.23) microns, respectively. The repeated measures analysis of variance showed that the three-year changes in different corneal regions were statistically significant (*p* < 0.001).

**Table 2:** The mean and 95% confidence interval (CI) of corneal thickness in center and different corneal regions according to two phases of Shahroud schoolchildren eye cohort study and sex and age groups.

Independent Variables		Central		Superior		Inferior		Nasal		Temporal	
		Phase 1	Phase 2	Phase 1	Phase 2	Phase 1	Phase 2	Phase 1	Phase 2	Phase 1	Phase 2
		Mean (95%CI)	Mean (95%CI)	Mean (95%CI)	Mean (95%CI)	Mean (95%CI)	Mean (95%CI)	Mean (95%CI)	Mean (95%CI)	Mean (95%CI)	Mean (95%CI)
	Total	558 (556;559)	557 (556;559)	661 (660;663)	670 (669;672)	632 (631;634)	627 (625;628)	645 (643;646)	649 (647;650)	623 (621;624)	621 (619;622)
Sex	Male	560 (558;562)	558 (556;560)	664 (662;666)	672 (670;674)	635 (633;637)	627 (625;629)	646 (644;648)	648 (646;650)	625 (623;627)	621 (619;623)
	Female	555 (554;557)	556 (554;558)	659 (657;661)	668 (666;670)	630 (628;631)	626 (624;628)	644 (642;646)	649 (647;651)	621 (619;623)	621 (619;623)
Age at baseline (year)	6	559 (553; 564)	561 (555; 566)	657 (651; 663)	671 (665; 677)	633 (627; 638)	631 (625; 636)	644 (638; 650)	652 (645; 658)	623 (618; 628)	624 (619; 630)
	7	561 (558; 565)	562 (559; 565)	662 (658; 665)	673 (669; 677)	635 (632; 639)	631 (628; 635)	648 (645; 652)	655 (651; 658)	626 (623; 630)	625 (622; 629)
	8	558 (555; 561)	558 (555; 560)	660 (657; 663)	669 (666; 672)	632 (629; 635)	627 (624; 630)	646 (643; 649)	649 (646; 652)	623 (620; 626)	621 (618; 624)
	9	562 (558; 565)	560 (557; 564)	665 (661; 668)	673 (670; 677)	636 (633; 640)	629 (626; 633)	649 (645; 653)	651 (648; 655)	626 (623; 630)	624 (621; 627)
	10	556 (553; 559)	555 (552; 558)	660 (657; 663)	668 (665; 672)	631 (628; 634)	624 (621; 627)	642 (639; 646)	645 (642; 649)	621 (618; 624)	618 (615; 622)
	11	554 (552; 557)	554 (551; 557)	661 (659; 664)	668 (665; 671)	629 (627; 632)	623 (620; 626)	641 (638; 644)	644 (641; 647)	620 (617; 623)	618 (615; 621)
	12	553 (549; 557)	552 (549; 556)	663 (659; 667)	668 (664; 672)	628 (624; 632)	622 (619; 626)	641 (636; 645)	643 (639; 647)	619 (615; 623)	616 (613; 620)

Table 3 presents the changes in corneal thickness in different regions by sex and age groups. As seen in Table 3, the most remarkable changes were observed in the superior corneal thickness [8.63 µm (95% CI: 8.1 to 9.17)]. On the other hand, the inferior corneal thickness decreased by −5.75 µm (95% CI: −6.3 to −5.2) in three years. The CCT in 6-year-old children increased by an average of 2.1 µm after 3 years. These changes were decreasing with a gentle slope as age progressed, resulting in an average −0.87 µm of CCT decrease in 12-year-old children after three years. There was a similar pattern of three-year changes with age for the thickness of all corneal regions.

**Table 3:** The mean and 95% confidence intervals (CI) of 3 years changes of corneal thickness in center and different corneal regions according to the sex and age groups.

Independent Variables		Central	Superior	Inferior	Nasal	Temporal
		Mean (95%CI)	Mean (95%CI)	Mean (95%CI)	Mean (95%CI)	Mean (95%CI)
	Total	−0.58 (−0.94;−0.22)	8.63 (8.10;9.17)	−5.75 (−6.30;−5.20)	3.72 (3.24;4.20)	−1.92 (−2.44;−1.41)
Sex	Male	−1.88 (−2.34;−1.42)	7.72 (6.97;8.47)	−7.68 (−8.37;−7.00)	2.25 (1.65;2.85)	−3.53 (−4.21;−2.86)
	Female	0.84 (0.44;1.24)	9.63 (8.91;10.34)	−3.63 (−4.30;−2.97)	5.33 (4.72;5.94)	−0.16 (−0.77;0.46)
Age at baseline	6	2.10 (0.51;3.69)	13.90 (11.85;15.95)	−1.83 (−4.39;0.73)	7.71 (5.34;10.07)	1.37 (−0.95;3.69)
	7	0.46 (−0.21;1.13)	11.51 (10.42;12.6)	−3.76 (−4.95;−2.58)	6.25 (5.37;7.13)	−1.21 (−2.27;−0.15)
	8	−0.72 (−1.52;0.08)	9.19 (7.98;10.40)	−5.42 (−6.61;−4.23)	3.76 (2.56;4.95)	−1.56 (−2.70;−0.42)
	9	−1.20 (−1.89;−0.50)	8.52 (7.45;9.60)	−6.83 (−7.91;−5.74)	2.58 (1.66;3.50)	−2.37 (−3.42;−1.32)
	10	−1.05 (−1.83;−0.28)	8.23 (7.26;9.19)	−7.15 (−8.40;−5.91)	3.10 (2.21;3.99)	−2.53 (−3.79;−1.27)
	11	−0.67 (−1.31;−0.04)	6.69 (5.67;7.72)	−6.45 (−7.44;−5.47)	3.14 (2.16;4.11)	−2.22 (−3.19;−1.24)
	12	−0.87 (−1.77;0.02)	5.41 (4.15;6.66)	−5.55 (−7.12;−3.98)	2.41 (1.37;3.45)	−2.61 (−4.2;−1.01)

**Fig. 2:**
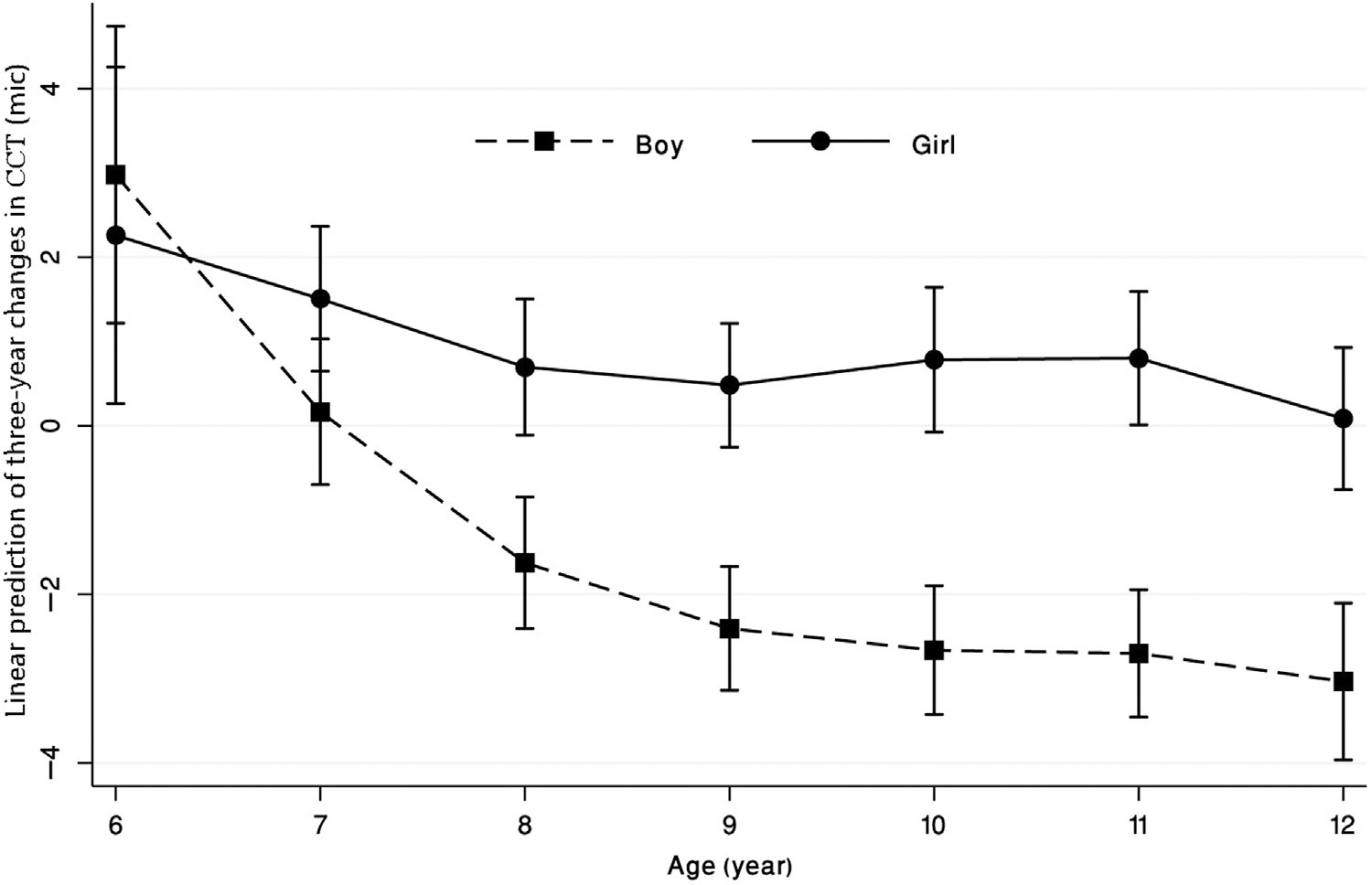
Predictive margins of the interaction between age and sex with 95% confidence intervals in three-year change of central corneal thickness (CCT) among schoolchildren between 6 and 12 years.

The mean three-year changes in CCT in males and females were −1.88 µm (95% CI: −2.34 to −1.42) and 0.84 µm (95% CI: 0.44 to 1.24), respectively; this difference was statistically significant (*p* < 0.001). However, there was a significant interaction between age and sex in corneal thickness changes (Fig. 2); major changes in the CCT occurred with age in boys while the age-related changes of this parameter in girls were almost negligible.

Table 4 shows the IQR, 95th, and 99th percentile of three-year changes in corneal thickness in the center and different regions of the cornea in the whole sample, distributed by age and sex. As seen in Table 4, the most remarkable changes occurred in the superior region with the 95th and 99th percentiles of 47 and 57 µm, respectively. In other words, 5% and 1% of the participants had increased thicknesses of more than 47 and 57 µm in the superior corneal region, respectively. Regarding the CCT, 5% and 1% of the children had an increased thickness of more than 20 and 25 µm, respectively.

**Table 4:** Interquartile range (IQR), 95th and 99th percentiles of 3 years changes of corneal thickness in center and different corneal regions according to the sex and age groups.

Independent Variables		Central	Superior	Inferior	Nasal	Temporal
		IQR;95%;99%	IQR;95%;99%	IQR;95%;99%	IQR;95%;99%	IQR;95%;99%
	Total	13; 20; 25	24; 47; 57	26; 28; 56	21; 35; 50	22; 27; 42
Sex	Male	12; 14; 20	23; 40; 61	22; 21; 38	19; 30; 45	21; 25; 42
	Female	11; 16; 22	22; 39; 57	21; 21; 37	19; 31; 45	21; 27; 41
Age at baseline	6	14; 19; 25	24; 43; 54	24; 27; 54	24; 36; 51	24; 31; 43
	7	13; 16; 21	24; 44; 68	24; 25; 49	21; 34; 49	23; 28; 42
	8	13; 15; 20	23; 40; 60	22; 23; 39	21; 31; 47	22; 27; 43
	9	12; 15; 22	21; 40; 57	21; 19; 34	19; 29; 42	22; 27; 43
	10	13; 14; 20	21; 38; 57	21; 18; 37	18; 29; 42	21; 25; 42
	11	11; 13; 21	20; 36; 53	19; 20; 34	17; 27; 41	19; 23; 38
	12	11; 13; 20	20; 33; 52	20; 19; 32	17; 27; 41	18; 21; 35

The association between 3-year changes in CCT with demographic and biometric variables was investigated using simple and multiple GEE models and the results are shown in Table 5. According to the multiple GEE model, there was a significant interaction between age and sex in the 3-year changes of the CCT, with boys experiencing a significant decline in CCT with advancing age. The multiple GEE model also showed that the three-year changes in the CCT were lower in rural than in urban students. Moreover, the baseline CCT, anterior chamber depth, and corneal diameter had a significant inverse association while the BMI and AL had a significant direct association with the three-year changes in CCT.

**Table 5:** Association of 3 years changes of central corneal thickness with demographic and ocular biometrics in simple and multiple generalized estimating equations models.

Independent variables		Simple model		Multiple model	
		Coefficient (95%CI)	p-value	Coefficient (95%CI)	p-value
Sex (Girl/Boy)	Girl	−0.74 (−3.44; 1.96)	0.591	−0.73 (−3.37; 1.91)	0.589
Age (year)	6	0		0	
	7	−2.87 (−4.83; −0.91)	0.004	−2.82 (−4.77; −0.87)	0.005
	8	−4.44 (−6.37; −2.52)	<0.001	−4.57 (−6.48; −2.65)	<0.001
	9	−5.09 (−7.00; −3.18)	<0.001	−5.39 (−7.29; −3.48)	<0.001
	10	−5.01 (−6.93; −3.09)	<0.001	−5.68 (−7.60; −3.75)	<0.001
	11	−4.68 (−6.59; −2.78)	<0.001	−5.68 (−7.61; −3.75)	<0.001
	12	−4.99 (−6.97; −3.01)	<0.001	−6.01 (−8.02; −4.00)	<0.001
Interaction between age and sex	Girl#6	0			
	Girl#7	2.43 (−0.52; 5.37)	0.107	2.06 (−0.82; 4.95)	0.161
	Girl#8	3.27 (0.37; 6.18)	0.027	3.00 (0.15; 5.86)	0.039
	Girl#9	3.68 (0.80; 6.57)	0.012	3.61 (0.78; 6.43)	0.012
	Girl#10	4.31 (1.40; 7.22)	0.004	4.20 (1.33; 7.07)	0.004
	Girl#11	4.02 (1.13; 6.91)	0.006	4.22 (1.37; 7.06)	0.004
	Girl#12	4.01 (1.05; 6.97)	0.008	3.84 (0.94; 6.74)	0.009
Residence place (Rural/urban)	Rural	−1.13 (−1.68; −0.58)	<0.001	−1.71 (−2.28; −1.13)	<0.001
Central corneal thickness at baseline (mic)		−0.04 (−0.05; −0.03)	<0.001	−0.04 (−0.05; −0.04)	<0.001
Height at baseline (Cm)		−0.01 (−0.03; 0.01)	0.166		NR
Weight at baseline (Kg)		0.01 (−0.02; 0.03)	0.662		NR
BMI at baseline (Kg/m^2^)		0.06 (−0.01; 0.13)	0.100	0.12 (0.05; 0.19)	0.001
Axial length at baseline (mm)		−0.74 (−1.04; −0.44)	<0.001	0.84 (0.45; 1.23)	<0.001
Mean keratometry at baseline (Diopter)		0.25 (0.09; 0.41)	0.003		NR
Anterior chamber depth at base line (mm)		−2.51 (−3.45; −1.57)	<0.001	−1.56 (−2.67; −0.45)	0.006
Lens thickness at base line (mm)		0.84 (−0.37; 2.04)	0.173		NR
Corneal diameter (mm)		−1.32 (−1.83; −0.82)	<0.001	−1.18 (−1.78; −0.58)	<0.001
Refractive errors	Emmetropia	0			
	Myopia	1.92 (0.75; 3.09)	0.001		NR
	Hyperopia	0.96 (−0.03; 1.96)	0.058		NR

CI, Confidence intervals; NR, Not retained in final model.

**Fig. 3:**
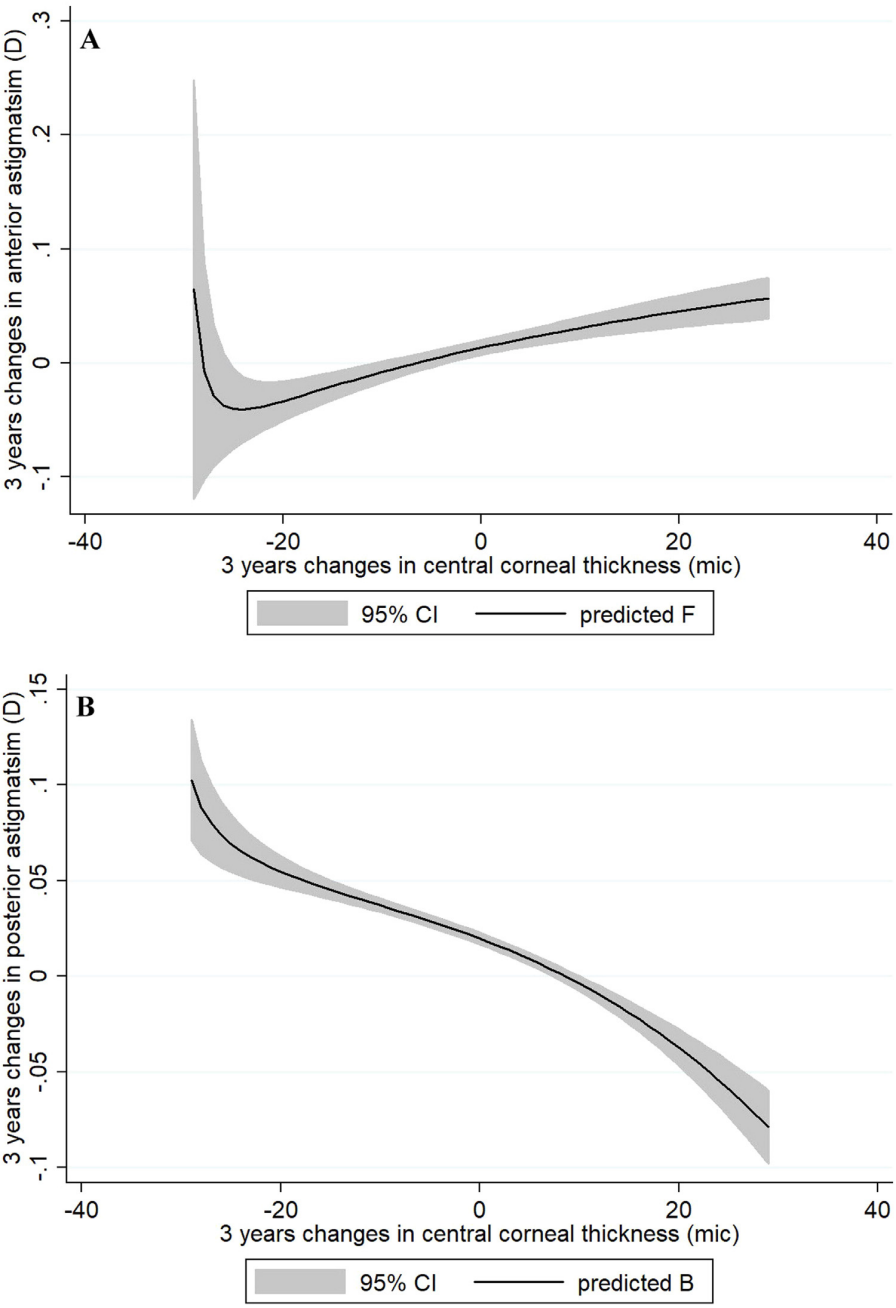
The anterior (A) and posterior (B) corneal astigmatism changes with alteration of central corneal thickness.

The study further investigated the association between changes in anterior and posterior corneal astigmatism and variations in CCT. Utilizing a multiple GEE model while controlling for age and sex, the results revealed a positive relationship between changes in anterior astigmatism and alterations in CCT (β = 0.002 95%CI: 0.001 to 0.002). Specifically, the data indicated that an increase in CCT corresponded with an elevation in anterior astigmatism (Fig. 3). Conversely, the analysis uncovered an inverse correlation between changes in posterior corneal astigmatism and CCT (β = −0.002 95%CI: −0.002 to −0.001)., indicating that as CCT increased, posterior astigmatism tended to decrease (Fig. 3).

## Discussion

During this three-year longitudinal study, the mean CCT in schoolchildren aged 6–12 years decreased by −0.58 µm (range: −0.94 to −0.22), although this decrease is not clinically significant and could be within the inter-examination variance of the Pentacam device. Knowing the pattern of corneal thickness changes in children would help in identifying ectatic abnormalities at an earlier age and recommending efficient interventions. Various studies have reported inconsistent results regarding the age-related changes in CCT in children. A prospective case series study revealed a mean CCT change of −1.9 ± 14 µm in children aged 3–14 years over a one-and-a-half-year period.^27^ This finding is about three times bigger than ours; however, this change was not statistically significant, possibly due to the small sample size (69 eyes), and the authors interpreted it as the stability of the CCT in children in the age range tested. When examining 12-year-old and 6-year-old children, a cross-sectional study indicated a decrease of 8.97 µm in the CCT^20^ Hussein et al.^28^ found an increase in the CCT with age; although they recruited a wider age range from 6 months to 18 years and reported that the CCT reaches an adult level at 5–9 years of age. The Pediatric Eye Disease Investigator Group study^29^ revealed that CCT increased from 1 to 11 years, but then remained unchanged until 17 years old. Other studies; however, reported no significant change in the CCT in children between 7 and 18 years of age.^19^^,^^30^^,^^31^ Although there is no clear explanation for the controversy in the literature, the results could be influenced by various age ranges, sample sizes, and CCT measurement devices and techniques.

The amount of change observed in this study was clinically negligible over three years in schoolchildren. Furthermore, a higher baseline CCT was linked to a smaller change in the CCT over the three years. As children aged, the amount of change in the CCT decreased, regardless of the direction of change. This may be due to the appearance of CCT stability in children between 6 and 12 years old. In line with this finding, the CCT was observed to be stable in a study by Muir et al. on children aged 3–14 years; however, their sample size was much smaller than ours. Different studies on children have indicated different ages for CCT to reach the adult level. For instance, Hussein et al.^28^ reported this age between 5 and 9 years, while the PEDIG study^18^ reported stability after 11 years old.

In line with other studies^19^^,^^32^, ^33^, ^34^ the thickest region of the cornea was the superior region, followed by nasal, inferior, and temporal regions in both phases of the study. Moreover, the corneal thickness in the superior and nasal regions of the cornea increased by significant amounts of 8.63 and 3.72 µm, respectively. The most significant change was seen in the superior corneal region. The underlying reason for this finding might be the anatomical changes in the vertical meridian to dampen against-the-rule astigmatism in school-aged children.^35^ It has been shown that the superior stroma exhibits a significantly higher interlamellar cohesive strength compared to the inferior stroma.^36^ The increase in superior corneal thickness may also result from hypoxia associated with eyelid coverage of the superior cornea^37^ as well as significant changes in eyelid muscle tonicity during puberty.^37^ This study, to our knowledge, is the first to longitudinally evaluate changes in corneal thickness in both the center and peripheral portions, which prevents us from comparing our results to those of other studies.

The majority of previous studies have shown that boys have a thicker CCT than girls^20^^,^^19^^,^^38^ However, we assessed the pattern of change in the CCT between boys and girls. According to our findings, there was a significant difference in the amount and type of change between boys and girls. Boys experienced a significant decline in CCT, while girls displayed a stable pattern over three years, as illustrated in Fig. 2. The difference in CCT change patterns between girls and boys could be due to sex-related differences in ocular structure^20^ as well as earlier puberty in girls, which need to be investigated in future studies.

Regarding the residence place, we found a greater reduction in the CCT in rural than urban children. The current evidence suggests that urban children and adults have a thicker CCT than their rural counterparts.^20^^,^^39^^,^^40^ Consistently, Vijay et al.^39^ assessed the CCT in 6754 adults aged ≥40 years and reported a greater decrease in the CCT (per decade of life) in rural than in urban residents. The corneal collagen fibers and keratocytes in rural residents may be affected by longer exposure to outdoor environments, which could result in a higher decrease in CCT, as suggested by Hahn et al. for the reverse association between CCT and aging.^41^ However, this issue needs to be assessed in future studies.

The association between BMI and AL has previously been reported among schoolchildren, with students with higher BMI having longer AL and vitreous length.^42^ We observed that higher BMI and AL at baseline were associated with greater CCT changes (i.e. increase in CCT) in children 6–12 years.

Research examining the association between BMI and CCT in pediatric populations is notably scarce. In a study conducted by Ngozika E Ezinne, no significant association was identified between CCT and BMI in children.^43^ Conversely, the body of literature concerning adults is more extensive, with several cross-sectional studies indicating a statistically significant direct relationship between CCT and BMI.^44^, ^45^, ^46^

The direct correlation between BMI and CCT may provide an alternative perspective on the association between BMI and IOP.^47^^,^^48^ Research indicates that individuals with elevated BMI may exhibit higher IOP measurements, potentially attributable to an increase in intraorbital fat, which elevates episcleral venous pressure and reduces the outflow facility.^46^ Conversely, it has been observed that eyes with thicker CCT tend to have higher IOP levels.^46^ Therefore, we believe that the observed correlation between CCT and BMI is likely influenced by IOP.

In terms of the more pronounced variations in CCT among individuals with increased AL, longitudinal studies have yet to be conducted. Furthermore, a cross-sectional study involving children aged 7 to 15 years did not establish a link between AL and CCT.^30^ In adult populations, the majority of research has similarly failed to demonstrate a significant association between CCT and AL. Nevertheless, our study represents the first longitudinal investigation to reveal changes in CCT in relation to AL. We propose that the observed relationship may not indicate a direct causal link between these two factors; rather, it may be an aspect of the emmetropization process occurring within this age group. This finding suggests that in individuals with longer eyes, the emmetropization process may lead to a corneal flattening to compensate for myopic shift. Furthermore, existing studies indicate that flatter corneas tend to exhibit a thicker CCT.^49^^,^^50^

## Conclusion

The findings of current study demonstrate an average decline of −0.58 µm in the CCT over three years, which is clinically insignificant and could suggest stability in the CCT. However, rural students and those with deeper anterior chamber depth and larger corneal diameter demonstrated a more significant decrease in CCT after three years. Also, longer AL and higher BMI was linked to an increase in CCT. The present study investigated the changes in the corneal thickness during puberty. Understanding the CCT changes in this life period can help to detect corneal abnormalities and ectatic conditions earlier and to provide timely intervention in suspicious cases.

## List of abbreviations

SSCECS, Shahroud Schoolchildren Eye Cohort Study; SD, Standard deviation; CI, Confidence intervals; CCT, Central corneal thickness; GEE, Generalized estimating equations; AL, Axial length; BMI, Body mass index; BCDVA, Best corrected distance visual acuity; UCDVA, Uncorrected distance visual acuity.

## Ethics approval and consent to participate

The study was approved by the Ethics Committee of Shahroud University of Medical Sciences (Reference Number: IR.SHMU.REC.1396.195). The study adhered to the tenets of the Helsinki Declaration at all stages. Prior to enrollment, the goals and methods of the study were explained to the parents in the presence of the students. We obtained written informed consent from all parents or students’ legal guardians and also oral consent from all the students.

## Consent for publication

Not applicable.

## Availability of data and materials

The data will be available in case of reasonable request by corresponding author.

## Funding

Shahroud School Children Eye Cohort Study is funded by the Noor Ophthalmology Research Center and Shahroud University of Medical Sciences (Grant Numbers: 960351).

## Author contributions

Design of study (MHE, HH, MK, and AF); Data collection (MHE, AF); Analysis and interpretation of data (AF, MK, EA and MHE); Writing of article (HH, EA, and MK); Critical revision and final approval of article (all authors).

## Financial support

Shahroud School Children Eye Cohort Study is funded by the Noor Ophthalmology Research Center and Shahroud University of Medical Sciences (Grant Number: 960351).

## Declaration of conflicting interests

The authors declare that there is no conflict of interest.
